# Impact of Health Labels on Flavor Perception and Emotional Profiling: A Consumer Study on Cheese

**DOI:** 10.3390/nu7125533

**Published:** 2015-12-09

**Authors:** Joachim J. Schouteten, Hans De Steur, Sara De Pelsmaeker, Sofie Lagast, Ilse De Bourdeaudhuij, Xavier Gellynck

**Affiliations:** 1 Department of Agricultural Economics, Ghent University, Coupure links 653, Gent 9000, Belgium; Hans.DeSteur@UGent.be (H.D.S.); Sara.DePelsmaeker@UGent.be (S.D.P.); Sofie.Lagast@UGent.be (S.L.); Xavier.Gellynck@UGent.be (X.G.); 2 Department of Movement and Sport Sciences, Ghent University, Watersportlaan 2, Gent 9000, Belgium; Ilse.DeBourdeaudhuij@UGent.be

**Keywords:** consumer, emotion, cheese, expectations, salt, fat, light, label

## Abstract

The global increase of cardiovascular diseases is linked to the shift towards unbalanced diets with increasing salt and fat intake. This has led to a growing consumers’ interest in more balanced food products, which explains the growing number of health-related claims on food products (e.g., “low in salt” or “light”). Based on a within-subjects design, consumers (*n* = 129) evaluated the same cheese product with different labels. Participants rated liking, saltiness and fat flavor intensity before and after consuming four labeled cheeses. Even though the cheese products were identical, inclusion of health labels influenced consumer perceptions. Cheese with a “light” label had a lower overall expected and perceived liking compared to regular cheese. Although cheese with a “salt reduced” label had a lower expected liking compared to regular cheese, no lower liking was found when consumers actually consumed the labeled cheese. All labels also influenced the perceived intensities of the attributes related to these labels, e.g., for example salt intensity for reduced salt label. While emotional profiles of the labeled cheeses differed before tasting, little differences were found when actual tasting these cheeses. In conclusion, this study shows that health-related labels might influence the perceived flavor and emotional profiles of cheese products.

## 1. Introduction

The frequency of heart disease and hypertension is increasing throughout the world and one of the reasons is a shift towards a more unbalanced diet, which includes a higher salt and fat intake [1,2,3]. Salt intakes in most high-income countries far exceed the upper limit of 5 g/day defined by the World Health Organization (WHO) [4]. As high salt intakes are linked to high blood pressure, the leading risk factor for early death [5], reducing salt intakes is seen as one of the most worthy objectives for increasing public health worldwide [6]. Reducing dietary fat intake gathered scientific interest in the last decade, as it is energy dense while having a rather limited effect on suppressing the appetite compared with protein or carbohydrate [7]. Its reduction may help lower the energy intake [8] and therefore prevent obesity which could lead to heart disease [9,10].

As a growing group of consumers are becoming more conscious with the health aspects of their diet [11,12,13], new food products have been developed which could address those needs and contain for instance less salt and fat. In order to better inform consumers of the improved composition and reformulation, these foods often contain front-of-pack labeling (*i.e.*, reduced in salt, “light”, *etc.*). Companies specifically target health-conscious consumers using such labels, which are potentially related to positive health outcomes (e.g., losing weight, lowering blood pressure, *etc.*) in the thoughts of consumers. However, one drawback is that consumers often associate changes in a particular ingredient, like salt reduction, with negative change of flavor. Liem *et al.* [14], for example, have found that the expected liking of soup was lower when the package also referred to salt reduction. A similar conclusion was made in a milk chocolate experiment where the expected liking decreased for “reduced-fat milk”-labeled products [8]. While these and other studies (for a review, see Fernqvist and Ekelund [15]), have shown that health-related claims could influence consumers liking of food products, it still remains to be investigated how the presence of such labels affects consumers’ expectations and actual experience of more specific sensory attributes. Because the acceptance of food products with health-related labels are also known to correspond with consumers’ attitudes and beliefs of such food products [16,17], it is crucial to understand the impact of health-related labels on consumers flavor perceptions in order to effectively promote healthy behavior.

From a theoretical point of view, three concepts are important about the potential influence of labels on the subsequent perception: (1) priming; (2) expectation theory; and (3) halo effect. The *priming theory* is initially developed in cognitive psychology [18,19] and comprises two phases. Participants are exposed to a stimulus (also known as prime), which can belong to any sensory modality (e.g., olfactory, visual, auditory, and flavor) during the first phase. The exposure to the prime leads to the activation of mental representations of the prime [20]. In a second phase, the unconscious effects are then evaluated as it is suggested that cues or primers can lead to the automatically activation of associated representations in memory increasing their accessibility [21,22]. As a consequence, Chambaron, *et al.* [22] state that exposure to a food-related stimulus (e.g., odor or message) may have important effects on subsequent eating behavior. Recent literature even suggest that priming with, for instance, fruit advertisements could improve the healthiness of food choices [21]. *Expectations research* has been widely applied in the field of food sensory and consumer research and examines the influence of information cues and expectations of those cues on the evaluation of food products. When one consumes a food or beverage, there may or may not be a disparity between the expected and actual experience. If such discrepancy occurs, a number of different outcomes could occur, as reported in previous literature [23,24], and four main psychological theories have been developed in order to explain such disconfirmation: (1) assimilation effect takes place when the participant adjusts his or her perception to what was expected, which results into the shift of product evaluation ratings in the direction of the participant’s prior expectations; (2) contrast theory can be applied when a person magnifies the difference which lead to the product evaluation ratings shifting into the opposite direction; (3) generalized negativity effect occurs when a consumer evaluates a product negatively because the expectations that they had prior to the evaluation were not met and therefore always lead to a lower product evaluation rating; and (4) assimilation/contrast theory depicts that assimilation will be observed if the disparity between the expected and experienced evaluation is rather small. If the discrepancy is too large, the contrast effect will likely occur instead. A recent review by Piqueras-Fiszman and Spence [25] contains a broad overview of research on sensory expectations with several types of information (including health-related information like nutrition content) and concludes that it is mostly the assimilation/contrast model that is applicable when testing food products with health-related information. Lastly, a *halo effect* could take place. The halo effect involves cognitive bias when the assessment of one particular characteristic (e.g., health label) of an item (e.g., food product) strongly affects the perception of other attributes (e.g., fat flavor perception and color intensity) of the same item [26]. An example is a recent study by Sütterlin and Siegrist [27] that found that using the label “fruit sugar” instead of “sugar” increased perceived healthiness of breakfast cereals.

It is also essential to measure beyond the overall acceptance of food products and obtain a broader perspective of consumers’ food product experience, given the high product failure rates at market introduction [28,29]. In the last years, assessing the emotional conceptualizations which consumers associate with food products have gathered momentum as a possibility to obtain additional information aside from the overall acceptance [30]. Several studies have illustrated that emotional conceptualizations can discriminate between food products even if the overall acceptance between products is similar [31,32,33]. Moreover, recent research suggests that including emotional measurements significantly improves food choice prediction of common acceptance measurements [34]. Thereby, emotions typically can be classified as “positive”, “unclassified” or “negative” [31,35], which could provide additional possibilities in further understanding consumer attitudes and beliefs towards food choice.

More recently, a growing number of studies have been carried out where consumers instead of the trained panelists performed sensory profiling of food products [36,37]. To ease and further improve the use of consumers for this kind of research, researchers have developed several new methodologies, such as check-all-that-apply, Napping^®^ and flash profiling [37,38]. Most new tools appear to be more cost efficient and allow retrieving direct feedback from consumers [37]. When compared with traditional profiling, these tools were successful at describing and quantifying product differences [37,38,39].

The purpose of the this study was twofold: (1) to examine the influence of potential health messages, like “reduced salt content” and “light”, on the expected and perceived sensory evaluation of cheese; and (2) to investigate which emotional conceptualizations consumers associate with such messages.

For this study, cheese was taken as a case. Cheese is an important source of dietary calcium, proteins and also vitamins [40,41,42]. Although cheese consumption is increasing worldwide [43], most cheeses have a rather high fat and salt content [42]. Therefore, new cheese products have been launched to address health conscious consumers for instance light cheeses (associated with a lower fat content) and low-sodium cheeses. Hence, this study aims to evaluate the effect of health-related labels on the expected and perceived flavor perception of cheese.

## 2. Material and Methods

### 2.1. Participants

Participants were recruited in Ghent area (Belgium) and no information about the aim of the study was provided at recruitment stage. Testing took place in the sensory facilities of Ghent University and consumers completed all the evaluations in sensory booths.

In total, 129 consumers participated in this test of which 53.4% were female. The mean participant age was 24.9 years (SD = 9.5), but participants ranged from 18 to 77 years. More than 80% of the participants ate cheese at least 2 to 3 times a week. Subjects were not compensated for their participation in the study.

### 2.2. Materials

#### 2.2.1. Cheese

Each participant received two pieces of one cheese at the same time (Boni selection Belgian young Gouda, purchased at Colruyt, Ghent, Belgium). All cheeses were exactly the same but different information was provided. A 3-digit random number was assigned to each sample and cheese slices were 1.5 cm × 1.5 cm × 1.5 cm [44]. Samples were served one at a time at 13 °C [45] on an odorless plastic plate.

#### 2.2.2. Health Labels and Experimental Design

To reduce the potential influence of a package (which includes, for example, brand and nutrient information), only a label description was included, in line with previous research on soy and organic labels [17,46]. The control label simply mentioned “cheese” (hereafter referred to as “control label”). The three other health-related labels were: (1) “cheese with reduced salt” (hereafter referred to as “reduced salt label”); (2) “light cheese” (hereafter referred to as “light label”); and (3) “light cheese with reduced salt” (hereafter referred to as “light + reduced salt label”). Cheeses containing these labels were available in major retailer stores across Belgium at the time of the study.

The presentation of the four labels were counterbalanced using a Williams design [47] to avoid confounds associated when using a within-participant design, such as first order and carryover effects. All consumers evaluated the four labels in both treatments).

### 2.3. Procedures

The tests were conducted in the sensory lab of the university. The respondents were told that they were going to evaluate four pieces of young Gouda cheeses and more detailed information about the cheese would follow when the evaluation started. This is comparable to the research of Liem, *et al.* [14], which worked with the same chicken soup while providing different health-related labels as information.

Before taking part in the study, potential participants were first required to complete a screening questionnaire in order to assess their suitability for the study. The screening criteria were based on their diet (consuming cheese products), food allergies (not lactose intolerant, no milk allergy or casein allergy) and their cheese consumption (at least once a month) [48,49].

**Figure 1:**
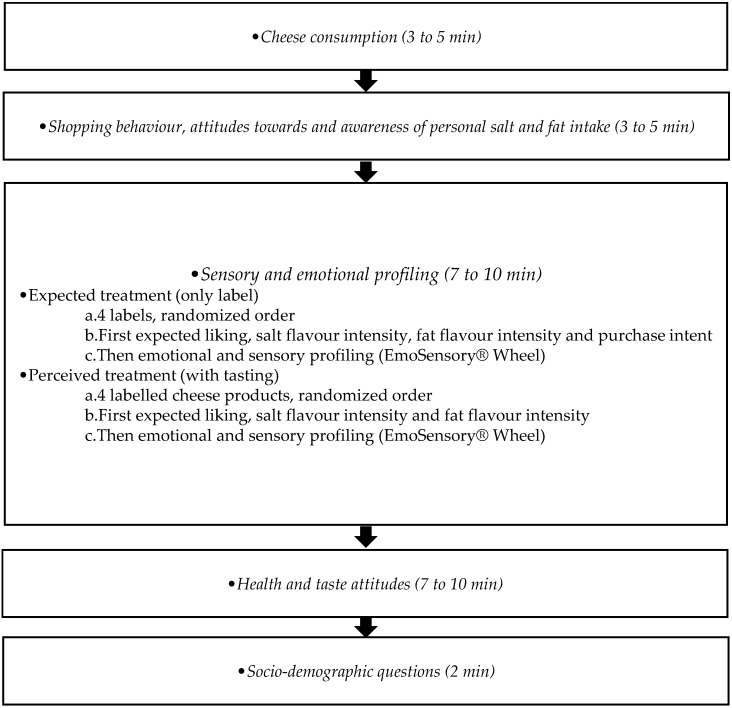
Questionnaire flow.

The questionnaire comprised five parts and was computer based using EyeQuestion v 3.12.0 software (Logic8 BV, Elst, The Netherlands) (Figure 1).

The first part examined the *consumption of cheese* with questions based upon a focus group discussion, prior research and reviewing the literature. The frequency of consumption was examined using 6 scale labels ranging from “daily” to “once a month”. Next, they indicated which type of cheese they consume followed by choosing their preferred type. Possible options were “hard cheese”, “soft cheese”, “cream cheese”, “light cheese”, “goat cheese”, “blue cheese” or “other”. If they chose “other”, they could specify their answer.

Questions regarding the participants’ *shopping behavior, attitudes* and *awareness* of *personal* *salt and fat* *intake* were asked in the second part of the study. This makes a more detailed classification of the sample possible. Regarding salt labeling, *three yes/no questions* were asked to assess *shopping behavior*, based upon previous research [50,51]. First, participants were asked “do you look for the salt content on food products when shopping?” Next, they were asked if salt content influenced purchases and if they often buy food products labeled as reduced salt products. Further *two items* reflecting *awareness of personal salt intake* were included. Participants were asked to which extend they have a diet with a low or high salt intake using a 5-point scale (“very low in salt”, “low in salt”, “average salt intake”, “high in salt”, “very high in salt”) [52]. To evaluate consumers’ salt intake, respondents were asked to compare their salt intake to the intake of men/women of the same age on a 5-point scale ranging from “much less” (1) to “much more” (5). *Intention to consume less salt* in their diet was asked using three possibilities: “no”, “yes, within 6 months” and “yes, within 1 month”. Finally, one question asked if participants thought that they need to have a diet low in salt on most days of the week using a 5-point Likert scale ranging from “strongly disagree” (1) to “strongly agree” (5) to reflect their *attitude towards salt consumption*. Similar questions were asked about the fat, e.g., “*Do you look for the fat content on food products when shopping*”, “*Does salt content influence your food purchases*” and “*Do you often buy low-fat labeled food products?*” Participants also reported to which extend their *diet contains fat* using a 5-point scale (“very low in fat”, “low in fat”, “average fat intake”, “high in fat”, “very high in fat”) [53,54]. Further, respondents were asked to *evaluate* their *fat intake* *compared* to a *men/women* of a *similar age* on a 5-point scale ranging from “much less” (1) to “much more” (5). Intention to consume less fat was questioned using the options “no”, “yes, within 6 months” and “yes, within 1 month”.

In the third part, participants’ *expectations* of the *salt intensity*, *fat flavor intensity* and *desire* of the four labeled cheeses were assessed. The labels were given in a random order to avoid order bias and carry-over effects [47]. Thereby, specific questions include: (1) How much do you think you will like the cheese; (2) “How salty do you think this cheese taste”; (3) “How fatty do you think this cheese will taste” and (4) “How much do you want to taste this cheese”. These questions were based upon Liem *et al.* (2012) and bipolar 7-point scales were used (*i.e.*, 1 = extremely dislike–7 = extremely like, 1 = not salty at all–7 = extremely salty, 1 = not fatty at all–7 = extremely fatty, 1 = do not want at all and 7 = want extremely). Recent work also suggests that fat flavor is a basic taste [55,56]. Further, consumers assessed the *emotional conceptualizations* and *sensory* *terms* that they associate with each cheese. Product specific emotional and sensory terms were determined during preliminary research following a two-step approach suggested by Ng, *et al.* [33] and Ares *et al.* [57]. First, a small group of consumers evaluated a list of emotional and sensory terms based upon previous studies [31,58,59,60,61,62,63]. The consumers also had the possibility to add their own terms and a focus group was held to see if additional terms were generated. Second, a final selection was made based on the number of people selecting the terms (≥15%) and the ability of the terms to discriminate between food products into account [29,35,64,65,66]. In addition, a balance between positive and negative emotions was made to easily compare the emotions and provide a global overview [35,58]. Sensory terms were selected to cover multiple sensory modalities (appearance, aroma, flavor, texture, aftertaste) [67]. An overview of the selected terms is listed in Table 1. As suggested in previous research with emotional terms [33], a rate-all-that-apply scale was used when the consumers evaluated the products during the consumer test. This scale has also been applied for the sensory profiling of several food products [67,68]. Participants used this scale to rate the intensity of the applicable sensory and emotional terms with a wheel format (EmoSensory^®^ Wheel, [69]) using a 5-point scale with end-point anchors 1 = “slightly” to 5 = “extremely”. Terms were given in alphabetical order, as this does not influence the results compared to a randomized presentation order [70,71].

After the participants expressed their expectations during the label only treatment, they received one cheese at the time to perform the perceived treatment. Participants were instructed to consume a first piece of cheese and rate (1) the overall liking; (2) salt flavor intensity and (3) fat flavor intensity of the cheese product using a 7-point bipolar scale (*i.e.*, 1 = extremely dislike–7 = extremely like, 1 = not salty at all–7 = extremely salty, 1 = not fatty at all–7 = extremely fatty). Next participants were asked to rate the intensity of the applicable sensory and emotional terms with the following instruction: “*Please try cheese sample XXX. Then, tick on each word that applies to describe cheese XXX and rate the intensity. Also, rate the intensity of applicable words which describe how you feel right now.*” This instruction was based upon previous work for the sensory [72] and emotional profiling [31] of food products. Lastly, consumers were asked to write down any remarks they had about the cheese products.

**Table 1:** Overview emotional and sensory terms.

Emotional Terms	Sensory Terms
Glad ^+^	Dry
Enthusiastic ^+^	Yellow
Irritated ^−^	Firm
Happy ^+^	Grainy
Good ^+^	Aftertaste
Calm ^u^	Pungent
Unpleasant surprise ^−^	Untasty
Discontented ^−^	Creamy
Disinterested ^−^	Soft
Dissatisfaction ^−^	Salty
Pleasant ^+^	Acid
Disappointed ^−^	
Merry ^+^	

^+, −, u^ means positive/negative/unclassified classified emotion.

In the next part, several statements were included to gain more information about the health and taste interests of the participants. As these statements are beyond the scope of this paper, this is mentioned for the sake of completeness but these are not discussed in further detail.

The last part contained several questions regarding the socio-demographic status of the respondents, such as age, gender, education level and place of residence.

### 2.4. Statistical Analyses

Repeated measures ANOVA and Bonferroni *post hoc* analyses were carried out to examine whether labels lead to different expectations regarding overall liking, salt intensity, fat flavor intensity and desire. The same analyses were performed after tasting the labeled cheese (perceived condition).

As suggested by Ares, *et al* [67], data obtained for the emotional and sensory characterization were analyzed using two different approaches, *i.e.*, frequency of selection or weighted frequency of selection (RATA scoring). RATA scorings take the actual points of the scale (ranging from 1 to 5) into account. Next, RATA scores for each emotional and sensory term were calculated by summing up the points. Cochran’s Q test was performed to determine significant differences in the frequency of term selection among the labels in both expected and perceived condition. Friedman’s test was carried out to identify significant differences in RATA scoring between the terms in either the expected or perceived condition. Further, repeated measures ANOVA with Bonferroni correction was performed to examine differences between the quantities of positive/negative emotions between the labels using sums of the frequency of term selection.

Power analysis was conducted using GPower 3.1 (Frans Faul, Kiel, Germany) [73] and tests that obtained a significant *p*-value (*p* ≤ 0.05) have a satisfactory power value above the threshold of 0.80. A 5% significance level (*p* ≤ 0.05) was considered for all tests, except when stated otherwise.

## 3. Results

### 3.1. Cheese Consumption, Shopping Behaviour, Attitudes and Awareness of Personal Salt and Fat Intake

#### 3.1.1. Cheese Consumption

Most participants consume cheese several times during a regular week. The participants are fond of hard and soft cheeses while a lot of the participants also consume goat cheeses. Light cheeses are only consumed by around a quarter of the sample. Almost half of the sample prefer hard cheeses, while around 15% of the sample prefer creamy and goat cheeses (Table 2).

**Table 2:** Cheese consumption and preferences of the sample (*n* = 129).

Consumption (%)		Consumption of Cheeses (%)		Preference (%)	
Once a month	3.9	Soft cheese	72.1	Soft cheese	11.6
Once a week	14.7	Hard cheese	88.4	Hard cheese	47.3
2 to 3 times a week	27.1	Creamy cheese	63.6	Creamy cheese	14.0
4 to 6 times a week	25.6	Light cheese	26.4	Light cheese	0.8
Daily	28.7	Goat cheese	67.4	Goat cheese	16.3
				Other	0.8

#### 3.1.2. Salt

**Figure 2:**
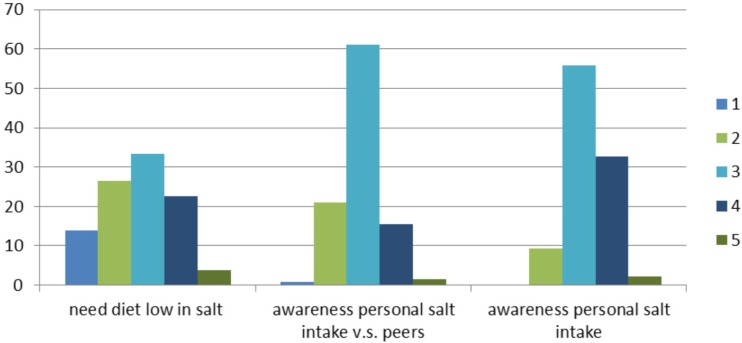
Respondents (in %) awareness of personal salt intake (very low in salt (1)–very high in salt (5)), awareness of personal salt intake compared with peers (consume much less salt (1)–consume much more salt (5)) and if they need a low-salt diet (totally disagree (1)–totally agree (5)).

Only 3% of the respondents state that they look to the salt content when buying products and a little bit over 2% declares that they often buy food products with a reduced salt content. A high majority (88.4%) of the sample does not see the salt content as a reason not to buy a food product. Given these numbers, unsurprisingly, over 90% of the participants are not planning to consume less salt in their diet. When asking for the consumers’ awareness of their salt intake, ranging from very low in salt to very high in salt, over half of the respondents answer that they have an average salt intake in their diet (Figure 2). If the participants need to compare their salt intake to those of their peers (same gender and similar age), most respondents answer that they consume similar salt intake like their peers. Lastly, more than one fourth of the participants find that they need to have a diet low in salt during most days in a week.

#### 3.1.3. Fat

**Figure 3:**
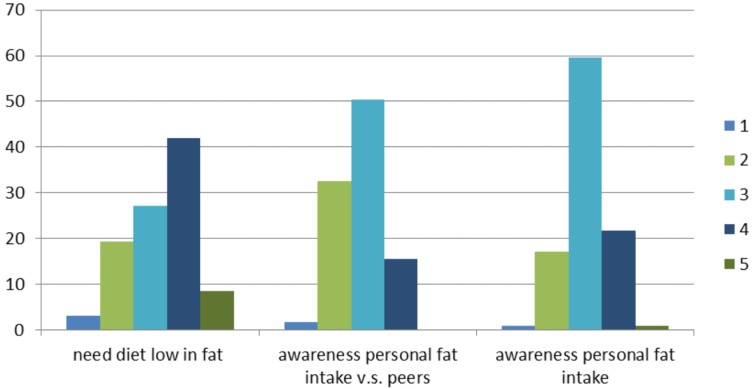
Respondents (in %) awareness of personal fat intake (very low in fat very high in fat (5)), awareness of personal fat intake compared with peers (consume much less fat (1)–consume much more fat (5)) and if they need a low-fat diet (totally disagree (1)–totally agree (5)).

Roughly one-third of the respondents (36.4%) declare that they have a look at the fat content when buying food products. Just over 51% of the participants declare that fat content can be a reason to not buy a certain food product. In addition, 31.8% of the respondents often buy low-fat products. In total, 30% of the participants are planning to consume more products with a lower fat content in the next six months. Even so, 17% of the respondents state that they are planning to consume fewer products with a lower fat content during the next month. Most participants estimate that their diet is rather average on fat intake and that the total fat intake is comparable with the mean intake of males/females of the same age (Figure 3). Almost half of the people find that they should have a low-fat diet on most days.

### 3.2. Liking, Salt Intensity, Fat Flavor Intensity and Desire

#### 3.2.1. Expected Condition

A significant main effect of labels on the expected liking (F(2.803,358.788) = 81.846, *p* < 0.001), salt intensity (F(2.712,347.167) = 101.478, *p* < 0.001), fat flavor intensity (F(3,384) = 90.889, *p* < 0.001) and desire (F(2.738,350.512) = 42.265, *p* < 0.001) was found.

**Figure 4:**
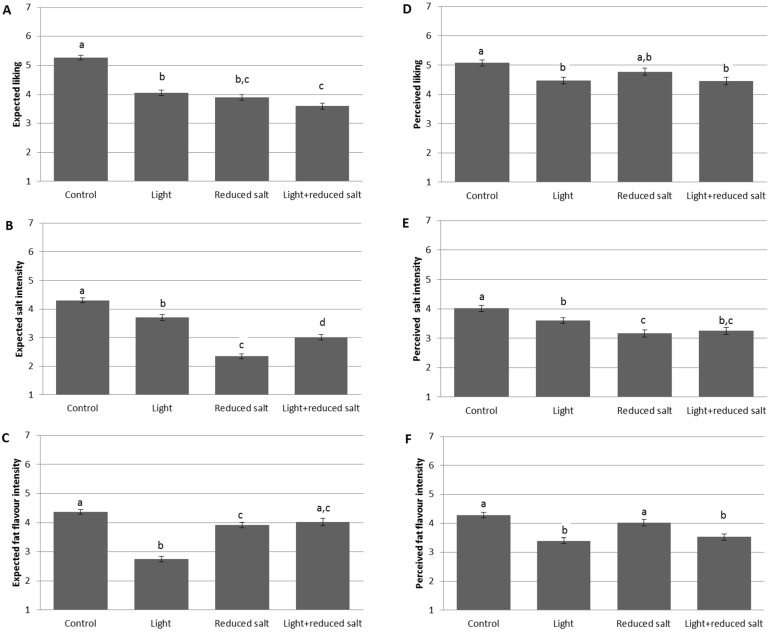
Expected liking (**A**); expected salt intensity (**B**); expected fat flavor intensity (**C**); perceived liking (**D**); perceived salt intensity (**E**) and perceived fat flavor intensity (**F**) of cheese with different labels (mean +SEM). Bars within a panel with the same letters do not differ significantly (*p* ≤ 0.05).

Figure 4A shows that the expected liking of the “control label” (5.26 ± 0.87) was significantly higher compared to the other labels. The mean expected liking of the “light label” (4.05 ± 1.14) was significantly higher compared to the “light + reduced salt” label (3.6 ± 1.18, *p* < 0.001), while it did not differ with the “reduced salt” label (3.9 ± 1.07, *p* = 1.0).

The expected salt intensity differed significantly among all labels (Figure 4B). The lowest mean salt intensity was expected with the “reduced salt” label (2.35 ± 0.97). The expected salt intensity was the highest for the “control label” cheese (4.30 ± 1.01). Overall, participants expected that the cheese with “light + reduced label” would have a salt intensity (3.01 ± 1.14) significantly higher compared to the “reduced salt label”(*p* < 0.001) but lower than the “light label” cheese (3.70 ± 1.14, *p* < 0.001).

The expected fat flavor intensity varied was significantly lower for the “light label” (2.75 ± 1.04) compared to the other three labels (Figure 4C). Participants expected that the fat flavor intensity of the “reduced salt label” cheese (3.91 ± 1.05) would be significantly lower compared to the regular, “control label” cheese (4.37 ± 0.89, *p* < 0.001). The mean expected fat flavor intensity of the “light + reduced salt” label cheese (4.02 ± 1.42) did not differ significantly between those latter two labels, but was significantly higher compared to the “light label” cheese (*p* < 0.001).

Consumers mainly show a desire for the “control label” cheese, as its expected desire was 5.16 ± 1.03. This expected desire was significantly higher compared to the other three labels (*vs.* light label 4.14 ± 1.32, *p* < 0.001; *vs.* reduced salt label 4.49 ± 1.24, *p* < 0.001; *vs.* light + reduced salt label 4.0 ± 1.42, *p* < 0.001). The mean expected desire for the “reduced salt label” cheese was significantly higher compared to the “light label” (*p* = 0.003) and “light + reduced salt label” (*p* < 0.001). No significant main effect of the label was found on the expected desire between the “light label” and the “light + reduced salt label” (*p* = 1.0).

#### 3.2.2. Perceived Condition

When participants evaluated the same cheese but provided with different labels, significant differences were found for the perceived liking (F(3,384) = 8.518, *p* < 0.001), salt intensity (F(3,384) = 16.655, *p* < 0.001) and fat flavor intensity (F(3,384) = 21.671, *p* < 0.001) (Table 3).

The highest perceived liking was for the cheese with the “control label” (5.07 ± 1.20), which was significantly higher compared to the “light label” (4.47 ± 1.32, *p* < 0.001) and “light + reduced salt label” (4.45 ± 1.42, *p* < 0.001). Consumers tend to like the “reduced salt label” (4.77 ± 1.35) as much as the “control label”, as no significant differences were found in the overall acceptance between both labels (*p* = 0.248). Further, the mean consumer liking between the “light label” and “light + reduced salt label” cheeses were very similar (*p* = 1.0) (Figure 4D).

When the cheese was provided with the “control label”, consumers tend to rate it saltier (4.04 ± 1.25) compared with when it had another label. If a “reduced salt label” was given, the perceived salt intensity (3.16 ± 1.41) was significantly lower compared to the “control label” (*p* < 0.001) and “light label” (3.60 ± 1.16, *p* = 0.012). There was no main effect of the label on the perceived salt intensity between the “reduced salt label” and “light + reduced salt label” (3.42 ± 1.42, *p* = 1.0). In addition, no significant effect of the labeling on the saltiness perception was found between the “light label” and “light + reduced salt label” (*p* = 0.072) (Figure 4E).

Regarding the perceived fat flavor intensity, the labels could be divided in two groups. When the cheese was provided with a “control label” (4.28 ± 1.13) or “reduced salt label” (4.02 ± 1.24), the perceived fattiness was significantly higher compared with the same cheese labeled as “light label” (3.40 ± 1.22) or “light + reduced salt label” (3.53 ± 1.17) (Figure 4F).

**Table 3:** Significant differences between expected and perceived treatment for liking, salt and fat flavor intensity. The levels for the ANOVA where the different labels (“control”, “light label”, “reduced salt label” and “light + reduced salt label”).

One-Way Repeated Measures ANOVA (*n* = 129)					
	*F*	*df*	*p*	Number of Levels	η^2^
*Expected liking*	81.846	2.803,358.788	<0.001	4	0.610
*Expected salt flavor intensity*	101.478	2.712,347.167	<0.001	4	0.728
*Expected fat flavor intensity*	90.889	3,384	<0.001	4	0.667
*Perceived liking*	8.518	3,384	<0.001	4	0.155
*Perceived salt flavor intensity*	16.655	3,384	<0.001	4	0.255
*Perceived fat flavor intensity*	21.671	3,384	<0.001	4	0.338

### 3.3. EmoSensory^®^ Characterization of the Labeled Cheeses

#### 3.3.1. Expected

**Figure 5:**
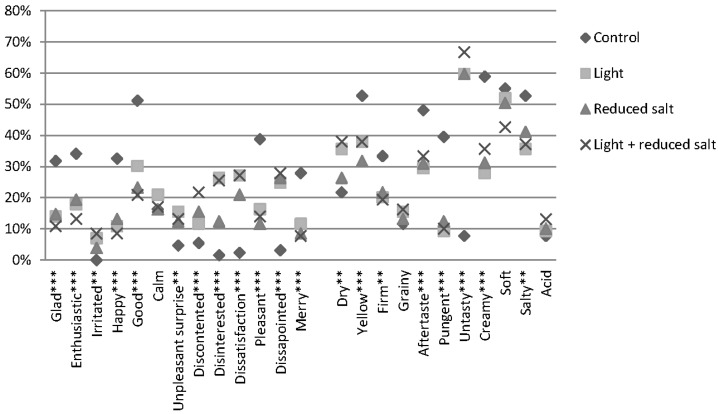
Expected EmoSensory^®^ profile of four labeled cheeses using the frequency count of selection. ** And *** indicate significant differences at *p* ≤ 0.01, and *p* ≤ 0.001, respectively.

Results related to frequency of use of both emotional and sensory terms are shown in Figure 5. Significant differences for the frequency of use between the differences labels were found for all emotions, except for the neutral emotion “calm” (*Q* = 1.892, *p* = 0.595). Consumers also expect differences on the sensory level between the different labeled cheeses as significant differences for eight out of 11 sensory attributes were found. When taking the actual scores into account for the analysis (RATA scoring), the same significant differences were found as when looking at the frequency of use. However, the RATA scoring approach lead to a higher significance level for three terms: “unpleasant surprise” (*p* < 0.001 *vs.* *p* = 0.001), “dry” (*p* = 0.001 *vs.* *p* = 0.004) and “salty” (*p* < 0.001 *vs.* *p* = 0.003).

If the valence of emotion (positive, negative or unclassified) is taken into account, the labels could be divided into three groups. The results show that the control label tends to be associated more with positive emotions like glad, happy and enthusiastic compared to the other labels. The other labels are also largely associated with negative emotions in both the frequency of use and rata scoring approach. A distinction between the reduced salt label and the two other labels (light label and light + reduced salt label) could further be made. Consumers have a more positive feeling about the reduced salt label compared to the other two labels, as can be seen in Figure 4. Repeated measures ANOVA revealed that there are indeed significant differences in association of positive (F(2.709,346.802) = 60.909, *p* < 0.001) and negative emotional conceptualizations (F(3,384) = 38.850, *p* < 0.001) between the labels. Consumers checked significantly more positive emotional terms with the control label (2.1) compared to the other three labels (reduced salt label: 1.0 (*p* < 0.001), light label (0.9, *p* < 0.001) and light + reduced salt label (0.8, *p* < 0.001)). No significant differences in the association of positive emotional terms were found between the three health-related labels. Consumers tend to associate almost no negative emotions to the regular cheese as a mean term selection of only 0.2 emotional terms was found. This was significantly less compared to the three health labels. The light + reduced salt label (1.2) had the highest association with negative emotions, which was significantly more compared to the “reduced salt label” (0.9, *p* = 0.011) but did not differ significantly with the “light label” (1.1, *p* = 1.0).

#### 3.3.2. Perceived

**Figure 6:**
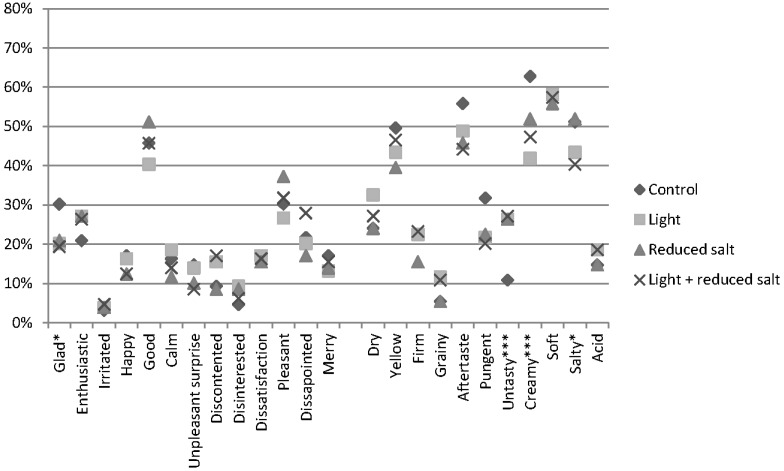
Perceived EmoSensory^®^ profile of four labeled cheeses. *, **, *** indicates significant differences at *p* ≤ 0.05, and *p* ≤ 0.001, respectively.

When consumers actually consumed the labeled cheese, few significant differences were found in the association with emotional and sensory terms (Figure 6). Significantly more consumers find the emotional term “glad” applicable to the control label, compared to the health related labels. Surprisingly, although consumers evaluated the same cheese but accompanied with different labels, significant differences in term usage were found for the sensory terms “creamy” (*Q* = 18.290, *p* < 0.001), “salty” (*Q* = 8.946, *p* = 0.030) and “untasty” (*Q* = 15.707, *p* = 0.001). Analyzing the data using the RATA scoring approach revealed additional differences in the sensory perception of the evaluated labeled cheese. Consumers perceived differences in the intensity of the “aftertaste” (χ^2^ (3) = 7.994, *p* = 0.046) and “yellow” (χ^2^ (3) = 15.060, *p* = 0.002) between the four samples of labeled cheese. Regarding the emotional terms, only a significant difference was reported for the emotion “glad” when taken the intensity into account. Regarding the type of emotions associated with the labeled cheeses, no significant differences were found for either positive (F(3,384) = 0.607, *p* = 0.611) or negative (F(3,384) = 0.976, *p* = 0.404) emotional terms. An overview of the differences in the emotional and sensory profiles is listed in Table 4.

**Table 4:** Summary of the differences of emotional and sensory terms during expected and perceived (with tasting) evaluation.

Cochran’s Q Test (RATA) and Friedman Test (RATA Scoring) (*n* = 129)	
*Expected Evaluation*	
Emotional terms with significant differences between samples	RATA: disappointed ***, discontented ***, disinterested ***, dissatisfied ***, enthusiastic ***, glad ***, good ***, happy ***, irritated ***, merry ***, pleasant ***, unpleasant surprise **
	RATA scoring: disappointed ***, discontented ***, dissatisfied ***, enthusiastic ***, glad ***, good ***, pleasant ***, happy ***, irritated **, merry ***, unpleasant surprise **
Sensory terms with significant differences between samples	RATA: aftertaste ***, dry **, creamy ***, firm **, pungent ***, salty **, untasty ***, yellow ***
	RATA scoring: aftertaste ***, dry ***, creamy ***, firm *, pungent ***, salty ***, untasty ***, yellow ***
*Perceived evaluation*	
Emotional terms with significant differences between samples	RATA: glad *
	RATA scoring: glad ***
Sensory terms with significant differences between samples	RATA: creamy ***, salty *, untasty ***
	RATA scoring: aftertaste *, creamy ***, salty **, untasty ***, yellow **

*, **, *** indicates significant differences at *p* ≤ 0.05, *p* ≤ 0.01, and *p* ≤ 0.001, respectively.

## 4. Discussion

This study illustrated that health labels can influence consumers’ flavor expectations of cheese. Several studies have found that health-related information like fat content [8,74,75,76], salt content [14], health logo [14], cholesterol reducing [77] and nutrition labels [76,78,79] could alter consumers’ expected acceptance of food products. In the current study, the expected liking of any health label (light or reduced salt or light + reduced salt label) was significantly lower compared to the control label cheese. Although food producers are using front of packaging labeling to communicate health-related credence attributes to consumers [80], they should be aware that taste oriented consumers could interpret these labels as a warning sign regarding their flavor [14]. Previous research suggest that a large group of consumers tend to associate healthy food with a lack of taste [81] and the use of specific health related labels like “light” could have a negative connotation and be more associated with “light in taste” than for instance “light in fat content” [82]. This negative effect of health labels on the expected liking could discourage taste-oriented consumers to even try or buy the product [14]. The lower negative expectations towards the health-related cheeses are not only limited to the expected liking, salt intensity and fat flavor intensity but is also reflected in the emotions consumers associate with these labeled cheeses. To date, few studies have examined the inclusion of emotional measurements next to overall acceptance during an expected condition. This study found that participants associate more negative emotions to these labeled cheeses compared to the control labeled cheese during the expected condition and also less positive emotions compared to the control labeled cheese. The current study illustrates the added value of including emotional measurements next to overall liking during an expected condition as suggested by Spinelli, *et al.* [32].

When participants consumed the same cheese, significant differences were not only found for the overall liking but also on the level of salt intensity and fat flavor intensity. These results demonstrate that a health halo effect could occurred when providing health-related information and confirm previous research results that also describe a health halo effect [14,27,83]. A number of studies have concluded that health-related labels could influence acceptance of food products as discussed extensively in a review by Fernqvist and Ekelund [15]. But one should note that consumers did actually evaluated the same cheese product during this study which was also the case in the study of the study with chicken soup of Liem, *et al.* [14]. Although Liem, *et al.* [14] did not find any influence of health labels on the flavor perception, in contrary to their hypothesis, they point out that the differences in acceptance between the expected and informed conditions were rather low. The perceived scores for the light and light + reduced salt labeling are significant lower compared to the control label cheese. For these two labels, an assimilation effect occurs as participants liking tend to go in the direction of their expected liking. As only around one quarter of the consumer sample consumes light cheese, it could be that most consumers are not fond of light cheese or have rather negative experiences with light cheese (or light products in general). Further, no significant difference were found between the control labeled cheese and the reduced salt labeled cheese in the current study. This is in accordance with recent research of Czarnacka-Szymani and Jezewska-Zychowicz [43] who found that labels containing the salt content did not alter consumers’ acceptance of several salt reduced cheeses. It seems that the respondents of this study have a more positive attitude towards this label compared to the light label and light + reduced salt label when they actually consumed labeled cheese. One reason could be that participants are less familiar with the reduced salt label (which is suggested by the fact that only 3% looks to the salt content on a package) and a disconfirmation effect occurs. Participants may think that a reduced salt labeled cheese does not taste good which can be seen in the rather low expected acceptance in this study. When they actually consumed the cheese, it disconfirms these prior expectations and they tend to overcompensate as illustrated in another experiment with healthy labeled entrees and desserts with diet labels [84]. In the case of the reduced salt label, the observed disconfirmation effect is considered to be a contrast effect. Further, the results of the perceived condition should be seen in the light of the priming theory and the presented results suggest that health-related labels might be used as a prime to guide people to make more healthier food choices. However, one should note that recent research suggest that the potential effect of health-related priming depends on individual traits like educational level and hunger states [21], so one need to bear this in mind when interpreting our results. A more specific research design would be needed when aiming to examine real behavioral outcome effects (e.g., ad libitum intake) of using health labels as primers and this yields an interesting potential for future research. As only one out of fourteen emotional terms differed significantly during the tasting, this study illustrates that the health-label information has little impact when consumers are actually tasting the same product. Previous research has already found that differences in emotional profiles of food products are primarily sensory driven [29,31,32,33,66,85,86,87,88] and the current study support these findings. Nevertheless, it is remarkable to note that labeling also influences the flavor perception of the sensory attributes “creamy”, “salty”, and “untasty”. This confirms previous research where information altered the intensity perception of sensory attributes [89,90]. While “untasty” can be seen as a more hedonic oriented sensory attribute, “creamy” and “salty” are definitely linked to the health-related labels that were used in this study. Using those labels could possibly draw the attention of the participants to related sensory terms, which are then perceived differently. This is in line with earlier findings that health claims on the front of the package leads to the generation of more attribute-specific thoughts about the product by consumers [91].

This study has several limitations. First of all, one should note that the used sample is not representative for the Belgian population. However, the use of a convenience sample at the university has been applied in several studies and provides interesting insights. A future study could use a greater sample size in order to obtain more power for the statistical tests. The tests took place in a lab which could bias the results as it does not mimics the reality but it has the major advantage that the experiment could take place in a better controlled environment. It has been previously reported that the context could influence the sensory [92,93] and emotional profiling [58,94] of food products. It could be interesting for future research to actually conduct tests in a more realistic situation, e.g., a shopping situation in a grocery store or the use of a product during a home use test. This study used three health-related labels and one product. Further research is needed with other health-related labels and also other products to examine if they underpin the findings of this study. In addition, this study has opted to examine the influence of the label on the flavor perception by letting consumers evaluate the same product for each label. However, future research needs to be performed with products in which the health labels are actually different in flavor and determine the critical composition in order to have a good balance between flavor and overall acceptance.

The present study holds practical implications for the role of front-of-package labeling. These labels could influence the sensory expectations and perception of related sensory attributes like fat content and salt content. Further, these labels could impact the emotional conceptualization of a food product. However, these effects are label-specific and the use of a reduced salt label did not lower the overall liking of cheese. Therefore, specific health-related labels might be used as a marketing tool in order to target specific health oriented consumers and even yield potential for priming healthy food products.

## References

[1] Doyle M.E., Glass K.A. (2010). Sodium reduction and its effect on food safety, food quality, and human health. Compr. Rev. Food Sci. Food Saf..

[2] Hooper L., Summerbell C.D., Higgins J.P.T., Thompson R.L., Capps N.E., Smith G.D., Riemersma R.A., Ebrahim S. (2001). Dietary fat intake and prevention of cardiovascular disease: Systematic review. BMJ.

[3] Mente A., de Koning L., Shannon H.S., Anand S.S. (2009). A systematic review of the evidence supporting a causal link between dietary factors and coronary heart disease. Arch. Intern. Med..

[4] Monro D., Mhurchu C., Jiang Y., Gorton D., Eyles H. (2015). Changes in the sodium content of New Zealand processed foods: 2003–2013. Nutrients.

[5] Lim S.S., Vos T., Flaxman A.D., Danaei G., Shibuya K., Adair-Rohani H., AlMazroa M.A., Amann M., Anderson H.R., Andrews K.G. (2012). A comparative risk assessment of burden of disease and injury attributable to 67 risk factors and risk factor clusters in 21 regions, 1990–2010: A systematic analysis for the global burden of disease study 2010. Lancet.

[6] Drake S.L., Lopetcharat K., Drake M.A. (2011). Salty taste in dairy foods: Can we reduce the salt?. J. Dairy Sci..

[7] Egger G., Swinburn B. (1997). An “ecological” approach to the obesity pandemic. Br. Med. J..

[8] Norton J.E., Fryer P.J., Parkinson J.A. (2013). The effect of reduced-fat labelling on chocolate expectations. Food Qual. Preference.

[9] Guh D.P., Zhang W., Bansback N., Amarsi Z., Birmingham C.L., Anis A.H. (2009). The incidence of co-morbidities related to obesity and overweight: A systematic review and meta-analysis. BMC Public Health.

[10] Van Gaal L.F., Mertens I.L., de Block C.E. (2006). Mechanisms linking obesity with cardiovascular disease. Nature.

[11] Januszewska R., Pieniak Z., Verbeke W., de Pelsmaeker S., Delbaere C., Depypere F., Kuti T., Hegyi A., Dewettinck K., Gellynck X. (2012). Food choice questionnaire revisited in four countries. Does it still measure the same?. Appetite.

[12] Guerrero L., Guàrdia M.D., Xicola J., Verbeke W., Vanhonacker F., Zakowska-Biemans S., Sajdakowska M., Sulmont-Rosse C., Issanchou S., Contel M. (2009). Consumer-driven definition of traditional food products and innovation in traditional foods. A qualitative cross-cultural study. Appetite.

[13] Kühne B., Vanhonacker F., Gellynck X., Verbeke W. (2010). Innovation in traditional food products in Europe: Do sector innovation activities match consumers’ acceptance?. Food Qual. Preference.

[14] Liem D.G., Toraman Aydin N., Zandstra E.H. (2012). Effects of health labels on expected and actual taste perception of soup. Food Qual. Preference.

[15] Fernqvist F., Ekelund L. (2014). Credence and the effect on consumer liking of food—A review. Food Qual. Preference.

[16] Aaron J.I., Mela D.J., Evans R.E. (1994). The influences of attitudes, beliefs and label information on perceptions of reduced-fat spread. Appetite.

[17] Wansink B., Park S.B. (2002). Sensory suggestiveness and labeling: Do soy labels bias taste?. J. Sens. Stud..

[18] Schacter D.L. (1987). Implicit memory: History and current status. J. Exp. Psychol. Learn. Mem. Cogn..

[19] Tulving E., Schacter D.L. (1990). Priming and human memory systems. Science.

[20] Shiffrin R.M., Schneider W. (1977). Controlled and automatic human information processing: II. Perceptual learning, automatic attending and a general theory. Psychol. Rev..

[21] Forwood S.E., Ahern A.L., Hollands G.J., Ng Y.-L., Marteau T.M. (2015). Priming healthy eating. You can’t prime all the people all of the time. Appetite.

[22] Chambaron S., Chisin Q., Chabanet C., Issanchou S., Brand G. (2015). Impact of olfactory and auditory priming on the attraction to foods with high energy density. Appetite.

[23] Cardello A.V., MacFie H.J.H., Thomson D.M.H. (1994). Consumer expectations and their role in food acceptance. Measurement of Food Preference.

[24] Cardello A.V., MacFie H.J.H. (2007). Measuring consumer expectations to improve food product development. Consumer-Led Food Product Development.

[25] Piqueras-Fiszman B., Spence C. (2015). Sensory expectations based on product-extrinsic food cues: An interdisciplinary review of the empirical evidence and theoretical accounts. Food Qual. Preference.

[26] Apaolaza V., Hartmann P., López C., Barrutia J.M., Echebarria C. (2014). Natural ingredients claim’s halo effect on hedonic sensory experiences of perfumes. Food Qual. Preference.

[27] Sütterlin B., Siegrist M. (2015). Simply adding the word “fruit” makes sugar healthier: The misleading effect of symbolic information on the perceived healthiness of food. Appetite.

[28] Cardello A.V., Meiselman H.L., Schutz H.G., Craig C., Given Z., Lesher L.L., Eicher S. (2012). Measuring emotional responses to foods and food names using questionnaires. Food Qual. Preference.

[29] Thomson D.M.H., Crocker C., Marketo C.G. (2010). Linking sensory characteristics to emotions: An example using dark chocolate. Food Qual. Preference.

[30] Köster E.P., Mojet J. (2015). From mood to food and from food to mood: A psychological perspective on the measurement of food-related emotions in consumer research. Food Res. Int..

[31] King S.C., Meiselman H.L. (2010). Development of a method to measure consumer emotions associated with foods. Food Qual. Preference.

[32] Spinelli S., Masi C., Zoboli G.P., Prescott J., Monteleone E. (2015). Emotional responses to branded and unbranded foods. Food Qual. Preference.

[33] Ng M., Chaya C., Hort J. (2013). Beyond liking: Comparing the measurement of emotional response using essense profile and consumer defined check-all-that-apply methodologies. Food Qual. Preference.

[34] Dalenberg J.R., Gutjar S., ter Horst G.J., de Graaf K., Renken R.J., Jager G. (2014). Evoked emotions predict food choice. PLoS ONE.

[35] De Pelsmaeker S., Schouteten J., Gellynck X. (2013). The consumption of flavored milk among a children population. The influence of beliefs and the association of brands with emotions. Appetite.

[36] Meiselman H.L. (2013). The future in sensory/consumer research: Evolving to a better science. Food Qual. Preference.

[37] Moussaoui K.A., Varela P. (2010). Exploring consumer product profiling techniques and their linkage to a quantitative descriptive analysis. Food Qual. Preference.

[38] Varela P., Ares G. (2012). Sensory profiling, the blurred line between sensory and consumer science. A review of novel methods for product characterization. Food Res. Int..

[39] Worch T., Lê S., Punter P. (2010). How reliable are the consumers? Comparison of sensory profiles from consumers and experts. Food Qual. Preference.

[40] O’Neil C.E., Keast D.R., Fulgoni V.L., Nicklas T.A. (2012). Food sources of energy and nutrients among adults in the US: Nhanes 2003–2006. Nutrients.

[41] Keast D.R., Fulgoni V.L., Nicklas T.A., O’Neil C.E. (2013). Food sources of energy and nutrients among children in the United States: National health and nutrition examination survey 2003–2006. Nutrients.

[42] Lucas A., Rock E., Chamba J.-F., Verdier-Metz I., Brachet P., Coulon J.-B. (2006). Respective effects of milk composition and the cheese-making process on cheese compositional variability in components of nutritional interest. Le Lait.

[43] Czarnacka-Szymani J., Jezewska-Zychowicz M. (2015). Impact of nutritional information on consumers’ acceptance of cheese with reduced sodium chloride content. Int. Dairy J..

[44] Santillo A., Caroprese M., Ruggieri D., Marino R., Sevi A., Albenzio M. (2012). Consumer acceptance and sensory evaluation of Monti Dauni Meridionali Caciocavallo cheese. J. Dairy Sci..

[45] Hersleth M., Ueland Ø., Allain H., Næs T. (2005). Consumer acceptance of cheese, influence of different testing conditions. Food Qual. Preference.

[46] Lee W.-C.J., Shimizu M., Kniffin K.M., Wansink B. (2013). You taste what you see: Do organic labels bias taste perceptions?. Food Qual. Preference.

[47] Macfie H.J., Bratchell N., Greenhoff K., Vallis L.V. (1989). Designs to balance the effect of order of presentation and first-order carry-over effects in hall tests. J. Sens. Stud..

[48] Meilgaard M.C., Carr B.T., Civille G.V. (2006). Sensory Evaluation Techniques.

[49] Lawless H.T., Heymann H. (2010). Sensory Evaluation of Food: Principles and Practices.

[50] Grimes C.A., Riddell L.J., Nowson C.A. (2009). Consumer knowledge and attitudes to salt intake and labelled salt information. Appetite.

[51] Webster J.L., Li N., Dunford E.K., Nowson C.A., Neal B.C. (2010). Consumer awareness and self-reported behaviours related to salt consumption in Australia. Asia Pac. J. Clin. Nutr..

[52] North S.L., Neale R.J. (1995). Knowledge, attitudes and eating habits of teenagers with respect to salt in their diet. Br. Food J..

[53] Brug J., van Assema P., Kok G., Lenderink T., Glanz K. (1994). Self-rated dietary fat intake: Association with objective assessment of fat, psychosocial factors, and intention to change. J. Nutr. Educ..

[54] De Bourdeaudhuij I., Brug J., Vandelanotte C., van Oost P. (2002). Differences in impact between a family *versus* an individual-based tailored intervention to reduce fat intake. Health Educ. Res..

[55] Running C.A., Craig B.A., Mattes R.D. (2015). Oleogustus: The unique taste of fat. Chem. Senses.

[56] Keast R., Costanzo A. (2015). Is fat the sixth taste primary? Evidence and implications. Flavour.

[57] Ares G., Barreiro C., Deliza R., Giménez A.N.A., Gambaro A. (2010). Application of a check-all-that-apply question to the development of chocolate milk desserts. J. Sens. Stud..

[58] Desmet P.M.A., Schifferstein H.N.J. (2008). Sources of positive and negative emotions in food experience. Appetite.

[59] Thomson D.M.H., Crocker C. (2013). A data-driven classification of feelings. Food Qual. Preference.

[60] Laros F.J.M., Steenkamp J. (2005). Emotions in consumer behavior: A hierarchical approach. J. Bus. Res..

[61] Szczesniak A.S. (2002). Texture is a sensory property. Food Qual. Preference.

[62] Salles C., Dalmas S., Septier C., Issanchou S., Noël Y., Etiévant P., le Quéré J. (1995). Production of a cheese model for sensory evaluation of flavour compounds. Le Lait.

[63] Mcewan J.A., Moore J.D., Colwill J.S. (1989). The sensory characteristics of cheddar cheese and their relationship with acceptability. Int. J. Dairy Technol..

[64] Manzocco L., Rumignani A., Lagazio C. (2013). Emotional response to fruit salads with different visual quality. Food Qual. Preference.

[65] Ferrarini R., Carbognin C., Casarotti E.M., Nicolis E., Nencini A., Meneghini A.M. (2010). The emotional response to wine consumption. Food Qual. Preference.

[66] Ng M., Chaya C., Hort J. (2013). The influence of sensory and packaging cues on both liking and emotional, abstract and functional conceptualisations. Food Qual. Preference.

[67] Ares G., Bruzzone F., Vidal L., Cadena R.S., Giménez A., Pineau B., Hunter D.C., Paisley A.G., Jaeger S.R. (2014). Evaluation of a rating-based variant of check-all-that-apply questions: Rate-all-that-apply (RATA). Food Qual. Preference.

[68] Jaeger S.R., Ares G. (2015). Rata questions are not likely to bias hedonic scores. Food Qual. Preference.

[69] Schouteten J.J., de Steur H., de Pelsmaeker S., Lagast S., de Bourdeaudhuij I., Gellynck X. (2015). An integrated method for the emotional conceptualization and sensory characterization of food products: The EmoSensory^®^ Wheel. Food Res. Int..

[70] King S.C., Meiselman H.L., Carr B.T. (2013). Measuring emotions associated with foods: Important elements of questionnaire and test design. Food Qual. Preference.

[71] Ares G., Jaeger S.R. (2013). Check-all-that-apply questions: Influence of attribute order on sensory product characterization. Food Qual. Preference.

[72] Jaeger S.R., Chheang S.L., Yin J., Bava C.M., Gimenez A., Vidal L., Ares G. (2013). Check-all-that-apply (CATA) responses elicited by consumers: Within-assessor reproducibility and stability of sensory product characterizations. Food Qual. Preference.

[73] Faul F., Erdfelder E., Lang A.-G., Buchner A. (2007). G* power 3: A flexible statistical power analysis program for the social, behavioral, and biomedical sciences. Behav. Res. Methods.

[74] Engell D., Bordi P., Borja M., Lambert C., Rolls B. (1998). Effects of information about fat content on food preferences in pre-adolescent children. Appetite.

[75] Kähkönen P., Hakanpää P., Tuorila H. (1999). The effect of information related to fat content and taste on consumer responses to a reduced-fat frankfurter and a reduced-fat chocolate bar. J. Sens. Stud..

[76] Ebneter D.S., Latner J.D., Nigg C.R. (2013). Is less always more? The effects of low-fat labeling and caloric information on food intake, calorie estimates, taste preference, and health attributions. Appetite.

[77] Kihlberg I., Johansson L., Langsrud Ø., Risvik E. (2005). Effects of information on liking of bread. Food Qual. Preference.

[78] Bayarri S., Carbonell I., Barrios E.X., Costell E. (2010). Acceptability of yogurt and yogurt-like products: Influence of product information and consumer characteristics and preferences. J. Sens. Stud..

[79] Carrillo E., Varela P., Fiszman S. (2012). Effects of food package information and sensory characteristics on the perception of healthiness and the acceptability of enriched biscuits. Food Res. Int..

[80] Hawley K.L., Roberto C.A., Bragg M.A., Liu P.J., Schwartz M.B., Brownell K.D. (2013). The science on front-of-package food labels. Public Health Nutr..

[81] Verbeke W. (2006). Functional foods: Consumer willingness to compromise on taste for health?. Food Qual. Preference.

[82] Viaene J. (1997). Consumer behaviour towards light products in Belgium. Br. Food J..

[83] Gravel K., Doucet É., Herman C.P., Pomerleau S., Bourlaud A.-S., Provencher V. (2012). “Healthy”, “diet”, or “hedonic”. How nutrition claims affect food-related perceptions and intake?. Appetite.

[84] Wansink B., Ittersum K.V., Painter J.E. (2004). How diet and health labels influence taste and satiation. J. Food Sci..

[85] Chrea C., Grandjean D., Delplanque S., Cayeux I., le Calvé B., Aymard L., Velazco M.I., Sander D., Scherer K.R. (2009). Mapping the semantic space for the subjective experience of emotional responses to odors. Chem. Senses.

[86] Gibson E.L. (2006). Emotional influences on food choice: Sensory, physiological and psychological pathways. Physiol. Behav..

[87] Porcherot C., Delplanque S., Raviot-Derrien S., Calvé B.L., Chrea C., Gaudreau N., Cayeux I. (2010). How do you feel when you smell this? Optimization of a verbal measurement of odor-elicited emotions. Food Qual. Preference.

[88] Porcherot C., Delplanque S., Planchais A., Gaudreau N., Accolla R., Cayeux I. (2012). Influence of food odorant names on the verbal measurement of emotions. Food Qual. Preference.

[89] Stolzenbach S., Bredie W.L.P., Christensen R.H.B., Byrne D.V. (2013). Impact of product information and repeated exposure on consumer liking, sensory perception and concept associations of local apple juice. Food Res. Int..

[90] Vidal L., Barreiro C., Gómez B., Ares G., Giménez A. (2013). Influence of information on consumers’ evaluations using check-all-that-apply questions and sorting: A case study with milk desserts. J. Sens. Stud..

[91] Wansink B., Sonka S.T., Hasler C.M. (2004). Front-label health claims: When less is more. Food Policy.

[92] Edwards J.S.A., Meiselman H.L., Edwards A., Lesher L. (2003). The influence of eating location on the acceptability of identically prepared foods. Food Qual. Preference.

[93] Köster E.P. (2003). The psychology of food choice: Some often encountered fallacies. Food Qual. Preference.

[94] Porcherot C., Petit E., Giboreau A., Gaudreau N., Cayeux I. (2015). Measurement of self-reported affective feelings when an aperitif is consumed in an ecological setting. Food Qual. Preference.

